# Whole-heart first-pass myocardial perfusion imaging with non-rigid respiratory motion correction

**DOI:** 10.1186/1532-429X-15-S1-P176

**Published:** 2013-01-30

**Authors:** Johannes Schmidt, Lukas Wissmann, Robert Manka, Sebastian Kozerke

**Affiliations:** 1 Institute for Biomedical Engineering, University and ETH Zurich, Zurich, Switzerland; 2 Imaging Sciences and Biomedical Engineering, King's College London, London, UK; 3 University Hospital Zurich, Zurich, Switzerland

## Background

Assessment of myocardial perfusion plays an important role in the evaluation of coronary artery and ischemic heart disease [1,2]. Scan acceleration methods such as k-t PCA [3] have proven invaluable for increasing coverage and have facilitated 3D perfusion imaging [4]. However, the sensitivity to respiratory motion for high acceleration factors remains challenging [4]. Especially in clinical routine, long breathhold durations are not always feasible.

In this work, an iterative k-t PCA algorithm is proposed which corrects for nonrigid frame-to-frame motion based on regularization with motion corrected k-t training data and motion operators thereof. It is demonstrated that this approach permits artifact-free reconstruction of 3D perfusion data acquired in non-compliant patients.

## Methods

Three motion corrupted 3D data sets were taken from a study of patients with suspected or known coronary artery disease. Data was acquired with a 10-fold k-t undersampled, saturation recovery gradient-echo sequence on a 3T Philips Ingenia system (Philips Healthcare, Best, The Netherlands). Scan parameters were: TR/TE: 2.2/1 ms, flip angle = 15°, spatial resolution: 2.3x2.3x10 mm3, saturation prepulse delay: 150 ms, acquisition window: 210 ms, #dynamics: 30.

Two out of the three data sets presented here were obtained under pharmacological stress (adenosine: 140 μg/kg/min over 6 min). For reconstruction, an iterative k-t PCA algorithm was implemented with all dynamic frames being warped to a reference respiratory position by an additional spatial transformation before applying Fourier and PCA transform in the time domain. The deformation fields for warping were estimated by image registration of frame-by-frame SENSE reconstructed images. To avoid misregistration due to contrast uptake, a b-spline transformation model was constrained to affine motion for several predefined image regions, including heart, liver, chest wall, and back. These regions were defined by an atlas based on a segmented multi-slice survey. Motion corrected images were compared to standard k-t PCA images without motion correction.

## Results

**Figure 1:**
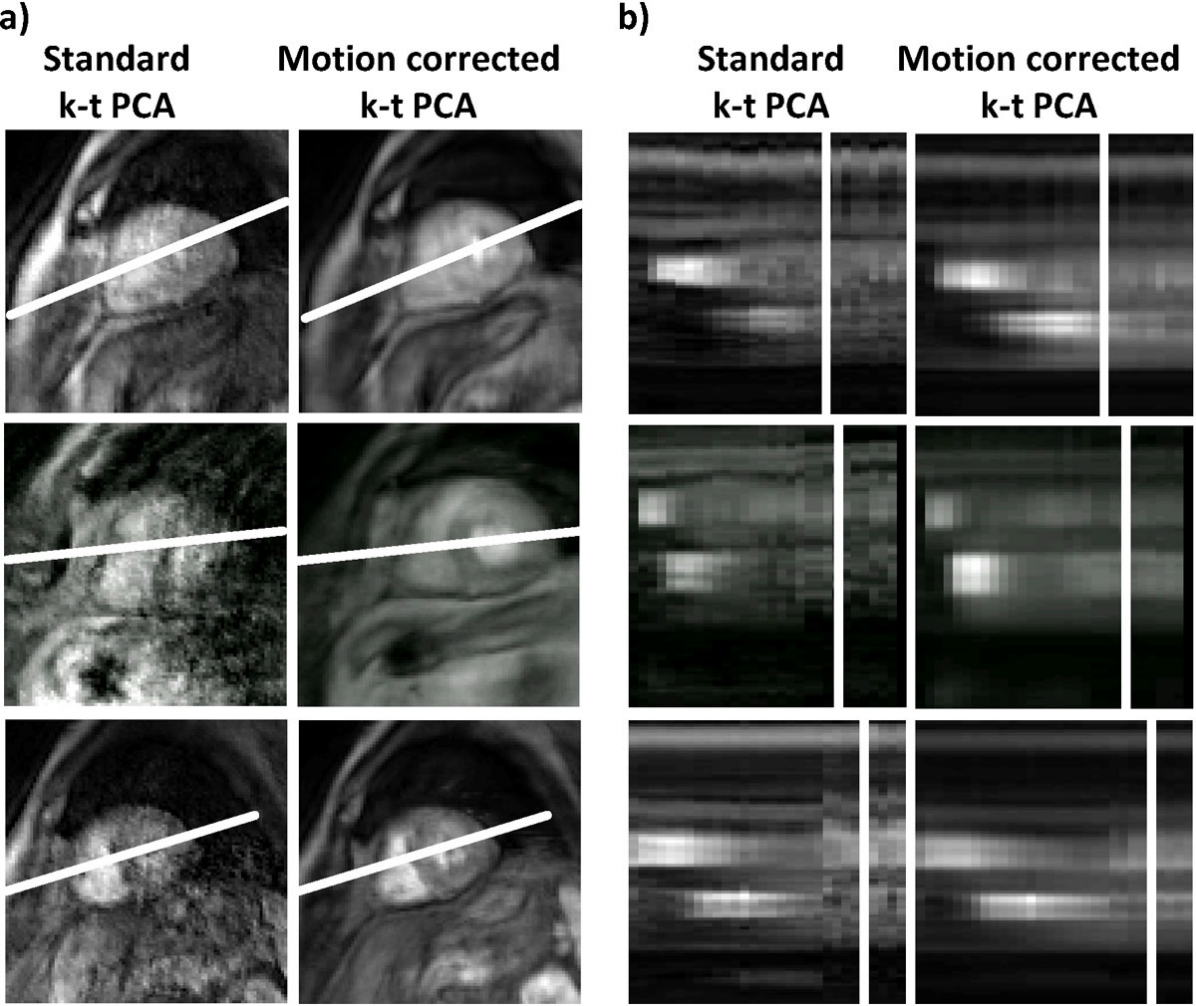
Image slices for a single time frame (a) as well as x-t profiles (b) reconstructed with standard k-t PCA and motion corrected k-t PCA.

**Figure 2:**
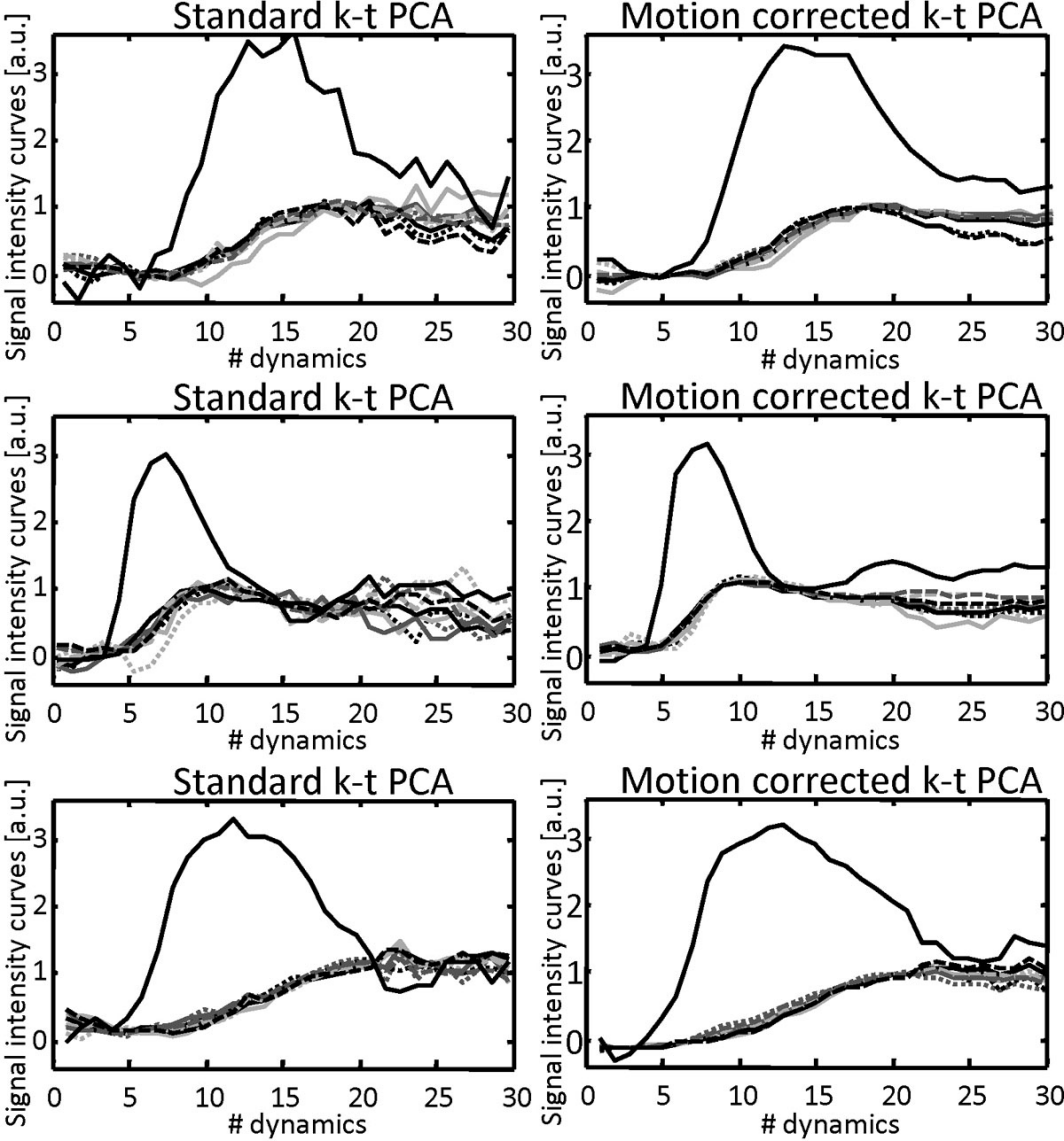
Signal intensity curves based on data reconstructed with standard k-t PCA (left) and motion corrected k-t PCA(right). The large solid black is from the left ventricular blood pool. Myocardial curves were extracted from a basal (light grey), equatorial (grey), and apical (black) slice.

Still frame images and profiles over time are shown for standard and motion corrected reconstruction in Figure 1. Image artifacts were successfully suppressed when using the motion-corrected k-t PCA implementation. In addition the approach allowed warping of all dynamic frames to a single respiratory position hence greatly simplifying image segmentation and analysis. Signal intensity profiles are shown for myocardium and left ventricular blood pool in Figure 2 demonstrating reduction of respiratory motion induced signal fluctuations with motion correction.

## Conclusions

Iterative k-t PCA with nonrigid motion correction enables correction of respiratory motion artifacts in dynamic 3D myocardial perfusion imaging. Thereby 3D perfusion data obtained during interrupted breathholding or even during free breathing can be successfully reconstructed and analyzed.

## Funding

The authors acknowledge funding by the Swiss National Science Foundation, grant #CR3213_132671/1 and by Bayer (Switzerland) AG.
